# Cinnamaldehyde, Carvacrol and Organic Acids Affect Gene Expression of Selected Oxidative Stress and Inflammation Markers in IPEC‐J2 Cells Exposed to *Salmonella typhimurium*

**DOI:** 10.1002/ptr.5705

**Published:** 2016-08-25

**Authors:** Sara A. Burt, Simone J.M. Adolfse, Dina S.A. Ahad, Monique H.G. Tersteeg‐Zijderveld, Betty G.M. Jongerius‐Gortemaker, Jan A. Post, Holger Brüggemann, Regiane R. Santos

**Affiliations:** ^1^ Institute for Risk Assessment Sciences, Division of Veterinary Public Health, Faculty of Veterinary MedicineUtrecht UniversityUtrechtThe Netherlands; ^2^ Biology Department, Faculty of ScienceUtrecht UniversityUtrechtThe Netherlands; ^3^ Department of BiomedicineAarhus UniversityAarhusDenmark; ^4^ Institute for Risk Assessment Sciences, Division of Veterinary Pharmacy, Pharmacotherapy and Toxicology, Faculty of Veterinary MedicineUtrecht UniversityUtrechtThe Netherlands; ^5^ Animal Sciences Post‐graduation ProgramFederal University of ParáBelémParáBrazil

**Keywords:** essential oils, gene expression, *Salmonella typhimurium*, IPEC‐J2, inflammation

## Abstract

Essential oils and organic acids are used as feed additives to improve health status and reduce colonization with pathogens. Although bactericidal *in vitro*, concentrations achieved in the animal gut are probably not lethal to pathogens. The aim of this study was to investigate the effects of cinnamaldehyde, carvacrol and cinnamic, lactic and propionic acids on the ability of *Salmonella typhimurium* ATCC 14028 (ST) to invade intestinal epithelial cells (IPEC‐J2) and on the expression levels of immune related genes in the cells. The minimum inhibitory concentration (MIC) and non‐inhibitory concentration (NIC) were determined and influence on the invasion capacity of ST was investigated. The structure of fimbriae and flagella was analysed by electron microscopy, and expression levels of *HSP70*, *IkBa*, *IL‐8* and *IL‐10* in the IPEC‐J2 cells were carried out by q‐PCR. Cinnamaldehyde, carvacrol and cinnamic and propionic acids inhibited ST invasion but not cell viability, bacterial viability and motility or the development of flagella. Propionic acid and cinnamaldehyde in combination with cinnamic acid caused structural impairment of fimbriae. Cinnamaldehyde up‐regulated expression of *HSP70* irrespective of the presence of organic acids or ST; exposure to carvacrol induced *HSP70* only in the presence of propionic acid and ST. © 2016 The Authors. *Phytotherapy Research* published by John Wiley & Sons Ltd.

## Introduction

*Salmonella typhimurium* is often isolated from pigs, and although farm animals do not always show clinical symptoms when colonized, in humans an infection with *S*. *typhimurium* can result in a serious gastrointestinal infection (EFSA, 2008). Reducing the number of food‐producing animals infected with salmonellae has been a goal of the animal production sector for many years (Altekruse *et al*., 1993; Fedorka‐Cray *et al*., 1997). Both plant‐derived essential oil compounds and organic acids are used as feed additives with the aim of reducing *Salmonella* colonization of food animals; in some cases, they have the added benefit of improving feed performance (Bravo *et al*., 2014; Creus *et al*., 2007; Kollanoor‐Johny *et al*., 2012; Walsh *et al*., 2012a, Walsh *et al*., 2012b). The antibacterial properties of some essential oil components are improved at low pH (Juven *et al*., 1994). Although essential oils and organic acids exhibit bactericidal properties *in vitro*, because of dilution in the gut and binding to protein and lipid components of the diet, the concentrations achieved in the animal gut are probably not lethal to pathogens. Any inhibitory effects on *Salmonella* colonization must therefore be because of other mechanisms, which have not yet been fully established (Inamuco *et al*., 2012; Upadhyaya *et al*., 2013). The plant‐derived compound carvacrol at sub‐lethal concentrations can induce heat shock proteins (HSPs) and inhibit the development of flagella in *Escherichia coli* (Burt *et al*., 2007). Carvacrol concentrations that do not inhibit bacterial growth can also inhibit motility in *S. typhimurium* and *Campylobacter jejuni* (Inamuco *et al*., 2012; Wieten *et al*., 2010). In addition to effects on bacteria, it may also be expected that plant compounds influence gene expression in intestinal cells (Inamuco *et al*., 2012). An overview of immune modulating effects of carvacrol has been provided by Alavinezhad and Boskabady (2014). Mechanisms for other potential therapeutic directions for carvacrol and cinnamaldehyde have also been reported (Gholami Mahtaj *et al*., 2015; Hong *et al*., 2016). Carvacrol‐containing herbs have long been used in ethnopharmacology, and its antimicrobial activity is probably the most useful of its properties (Guenther, 1948; Sajed *et al*., 2013). Cinnamaldehyde, present in cinnamon oil, also has antibacterial properties and useful activity when added to animal feed (Palaniappan & Holley, 2010; Shahverdi *et al*., 2007; Upadhyaya *et al*., 2013).

Short and medium chain fatty acids have been shown to reduce the colonization of porcine gut cells by Salmonellae and of the chicken gut by *Campylobacter* spp., and addition of acids to feed or water of pigs and poultry has become a common practice, particularly since the ban on the use of antimicrobial feed additives in the EU (Boyen *et al*., 2008; Van Gerwe *et al*., 2010).

Synergetic and antagonistic interactions between chemical compounds can strongly affect their function and their usefulness in practice (White *et al*., 1996). An overview of analytical methods for testing for interactions between essential oils and other compounds has recently been provided (Langeveld *et al*., 2014). An interaction is said to be synergistic when the combined effect is greater than the sum of the individual substances; when the combined effect is smaller than that of the individual substances, it is termed antagonism (Bhat & Ahangar, 2007). Synergy between compounds can be useful in the case of feed additives because if their combined effects are greater than the sum of the components, smaller amounts of both may be used, leading to lower costs. Antagonism between compounds is a hindrance and needs to be avoided if possible. As yet there are, however, little data on synergy testing between carvacrol, cinnamaldehyde and a number of organic acids that are suitable as feed additives.

The aim of this study was to investigate the effects of cinnamaldehyde, carvacrol and selected organic acids, individually and combined, on the ability of *S. typhimurium* to invade intestinal epithelial cells by looking at effects on the bacteria (fimbrial structure and flagellar function, protein synthesis) and on the epithelial cells (expression levels of immune related genes). For this study, we used the non‐transformed cell line IPEC‐J2 (intestinal porcine epithelial cells from jejunum) derived from jejunal epithelia of unsuckled piglets, which is polarized, non‐cancerous and has been characterized as a model for the porcine gut (Berschneider, 1989; Schierack *et al*., 2006).

## Materials and Methods

### Test compounds

The compounds used for the assays were as follows: carvacrol (98%), trans‐cinnamaldehyde (99%), cinnamic acid (99%), lactic acid and propionic acid (>99.5%), all obtained from Sigma‐Aldrich, Zwijndrecht, The Netherlands. Ethanol 98.6% *v/v* was used as a solvent unless otherwise specified. During method development, pilot experiments were carried out to check that the concentration of ethanol used in the experiments was not toxic to the bacteria or to the cell line. Nowhere was this higher than 1% *v/v*. Data given for the control treatment is control including solvent (ethanol), unless otherwise specified.

### Bacterial strains and culture conditions

*S. typhimurium* ATCC 14028 (ST) was maintained on tryptone soya broth agar slants at 4 °C (TSBA, Oxoid + 1% bacteriological agar). Bacteria were cultured overnight in Luria Bertani broth (LB, 1% tryptone, 0.5% yeast extract, 0.5% NaCl) at 37 °C with 150‐rpm shaking for 16 h. For invasion assays, a 200‐μL portion of 16‐h culture was transferred to 20‐mL LB with or without the addition of the test compounds and cultured with shaking to an optical density (OD, 550 nm) of 0.5, indicating the bacteria had reached early log phase (approx. 3 h). The OD of the culture was adjusted to achieve the required number of cfu/mL before each experiment.

### Determination of minimum bactericidal concentrations (MBC), minimum inhibitory concentrations (MIC), non‐inhibitory concentrations (NIC) and interactions between compounds

Portions of 100‐μL increasing concentrations of the chosen test compounds in LB were placed in 100 well microplates. Concentration ranges were: cinnamaldehyde 0 – 400 µg/mL, carvacrol 0 – 300 µg/mL, cinnamic acid 0 – 3000 µg/mL, lactic acid 0 – 3500 µg/mL and propionic acid 0 – 6000 µg/mL. One hundred microlitre aliquots of the bacterial suspension were added to achieve a bacterial density of 10^5^ cfu per well. The plates were incubated in an automated optical density reader (software version 2.28; Bioscreen, Oy Growth Curves AB Ltd, Helsinki, Finland) at 37 °C with shaking. The OD was measured at hourly intervals, producing a growth curve. After 20‐h incubation, the minimum inhibitory concentration (MIC) was determined as the lowest concentration at which no bacterial growth was measured. For determination of the minimum bactericidal concentration (MBC), 10‐μL portions from microplate wells showing no bacterial growth were plated out onto Luria Bertani agar (LBA, LB broth + 1% *w/v* bacteriological agar, Oxoid) and incubated for 24 h at 37 °C. The MBC was the lowest concentration at which no viable bacteria could be cultured. Further, the highest concentration at which no influence was seen on the growth curve compared to the untreated control was recorded as the NIC. Each experiment was carried out three times in duplicate.

To detect any synergy or antagonism which may exist between cinnamaldehyde or carvacrol and the acids, checkerboard assays were carried out whereby increasing concentrations of one compound were placed in the horizontal rows and increasing concentrations of the other in the vertical ‘columns’ of a microplate. After adding bacteria, plates were incubated in an OD reader as described above. After 20‐h incubation at 37 °C with shaking, the fractional inhibitory concentrations (FICs) were calculated from these results using the following formulae:

$$\begin{array}{l} FIC\left(A\right)\;=\;\frac{\text{MIC}\left(A\;\text{in the presence of}\;B\right)}{\text{MIC}\;\left(A\right)}\\ FIC\left(B\right)\;=\;\frac{\text{MIC}\;\left(B\;\text{in the presence of}\;A\right)}{\text{MIC}\left(B\right)}\\ \text{where}:\\ A:\text{represents cinnamaldehyde or carvacrol}\\ B:\text{represents the respective acids}\text{.} \end{array}$$

The FIC index for each combination was then calculated by averaging the FICs for both test compounds. The results were interpreted as synergistic when the FIC index for the combination was less than or equal to 0.5, as additive when the index was between 0.5 and 1.0, as indifferent when the index was between 1.0 and 2.0 and as antagonistic when the index was greater than 2.0 (EUCAST, 2000)

### Bacterial motility

Whether the test compounds had any influence on the motility of the selected bacterial strains was determined by growth on semi solid motility agar containing 1.0% *w/v* tryptone (BD Biosciences), 0.5% *w/v* sodium chloride and 0.3% *w/v* bacteriological agar (Oxoid). Appropriate amounts of stock solution of the test compounds were added to 20‐mL aliquots of molten agar to achieve increasing concentrations up to the corresponding MIC and the plates were allowed to set. A negative control was included in which the same volume of ethanol (solvent) as in the maximum concentration of test compound was added to the agar. The plates were dried by overnight incubation at 37 °C and inoculated with a 16‐h culture of bacteria by central stab inoculation using a sterile cocktail stick. The diameter of the area of motility was measured after 24‐h incubation at 37 °C. Experiments were carried out twice in duplicate, and mean diameters were calculated for the motility areas and presented graphically.

### Cell culture conditions

IPEC‐J2 cells were maintained in growth medium (Advanced DMEM/F12, Gibco) with added 0.2 mM L‐glutamine (Gibco) and 5% foetal bovine serum (FBS, Lonza) and incubated at 37 °C in an atmosphere containing 5% carbon dioxide and with a relative humidity of 86%. The assays used cells between passages 80 and 100. Cells were prepared for an assay by culturing to confluence in 12 wells plates and washing three times with warm plain medium.

### Cell viability assays

Two concentrations (the MIC and 0.5 × MIC) of each test compound were tested for possible effects on the viability of IPEC‐J2 cells using the trypan blue exclusion assay in which cells whose membrane has become permeable stain blue, whilst viable cells remain unstained. Cells were prepared for an experiment by culturing to 95% confluence in 12 wells plates and washing three times with Dulbecco's phosphate buffered saline (DPBS) with calcium and magnesium. The DPBS was then replaced by growth medium without added L‐glutamine and FBS and containing concentrations of test compounds corresponding to the MIC and 0.5 × MIC. After a 2‐h incubation period (37 °C, 5% CO2) cells were washed three times with DPBS without Ca/Mg and separated from the wells using a solution of trypsin‐EDTA (Lonza). After centrifugation of the content of the wells at 1000 rpm (300 rcf), the supernatant was reduced to approximately 100 μL by pipette and the cells resuspended by gentle vortexing. A 40‐μL portion of the resulting cell suspension was mixed with 20 μL of 0.4% trypan blue in physiological saline solution and within 5 min the numbers of viable and non‐viable cells in this suspension were counted using a Fuchs–Rosenthal haemocytometer observed through a phase contrast microscope at magnification 100×. Ethanol (solvent) was included as negative control. The percentage of viable cells after 2‐h exposure was calculated for the MIC and 0.5 × MIC for each test compound. Experiments were carried out three times in duplicate. The classification of viability was carried out by a colleague who did not prepare the samples (blinded).

### Bacterial adhesion and invasion assays

Confluent IPEC‐J2 monolayers were exposed to ST for a period of 1 h (37 °C, 5% CO2, 86% RH) in the presence or absence of the test compounds at a multiplicity of infection of 20:1 bacteria to host cells. Negative controls were exposed to plain culture medium and to medium containing only solvent (ethanol). Positive controls were exposed to bacteria only. In the first series of experiments, the concentrations of test compounds added to the cell culture medium was 0.5 × MIC, which was a concentration not toxic to the cells or the bacteria (Table 1). In a second series of experiments even lower concentrations were used, namely the highest concentration of compound which did not affect the growth curve of the bacteria compared to control, termed the NIC (Table 1).

**Table 1:** Minimum bactericidal concentration (MBC), minimum inhibitory concentration (MIC) and non‐inhibitory concentration (NIC) for all compounds

	Test compounds (µg/mL)				
	Cinnamaldehyde	Carvacrol	Cinnamic acid	Lactic acid	Propionic acid
MBC	387	231	2000	3000	5000
MIC	156	154	2000	3000	1000
0.5 × MIC	78	77	1000	1500	500
NIC	26	31	200	600	400

Lactic acid was not used in further assays because it showed the least effect on *Salmonella* invasion. For ST which can adhere to the host cell surface but infects cells by invasion, a distinction could be made between invaded (intracellular) bacteria and adherent bacteria. To determine the number of cell‐associated bacteria, cells in half the wells were washed three times with warm plain medium and lysed using 0.1% *v/v* Triton X‐100 (Sigma) to release intracellular bacteria. Serial tenfold dilutions of the resulting suspensions were plated out on LB agar and incubated at 37 °C for 20 h. Bacterial invasion was quantified in the other half of the wells by treating the cells with 300 µg/mL colistin (Gibco) for 2 h to kill externally adherent bacteria (37 °C, 5% CO_2,_ 86% RH) before lysing and plating out on agar. The number of intracellular bacteria was subtracted from the number of cell‐associated bacteria to calculate the number of adherent bacteria. Experiments were carried out three times in duplicate.

### Visualization of fimbriae and flagella by electron microscopy

ST possesses bristle‐like type 1 fimbriae which are 5 – 7 nm in diameter and about 1 µm long and flexible flagella which are about 20 nm in diameter and 15 – 20 µm long (Althouse *et al*., 2003). Bacteria cultured 16 h in the same concentration of compound used in the invasion assays (the NIC, Table 1) were prepared for electron microscopy as follows. The coated side of a copper grid, coated with 0.7% Formvar (Agar Scientific, Essex, UK), was incubated on a drop of bacterial culture for 4 min, allowed to dry in air, rinsed twice with distilled water, dried, stained 3 min with 2% uranyl acetate solution and dried again. Samples were observed under a FEI Tecnai 10 transmission electron microscope with a SIS (Olympus) Megaview II side entry 1 K camera and compared to untreated control cultures.

### Quantitative RT‐PCR

IPEC‐J2 monolayers either exposed or not exposed to invasion, in the presence or absence of test compounds, were washed three times with warm plain medium to remove non‐adherent bacteria and lysed by adding 175‐μL RNA lysis buffer (Promega) per well. Samples were snap frozen in liquid nitrogen and stored at −80 °C until PCR analysis. The experiment was carried out at least three times.

Six relevant stress and immune response‐associated genes were selected for analysis: a gene regulating cell stress like heat shock protein 70 (*HSP70*); genes regulating inflammatory response such as the inhibitor of nuclear transcription factor (*IkBα*), and interleukins 8 (*IL‐8*), 10 (*IL‐10*) and 12 (*IL‐12*). A ratio on the expression of *IL‐8/IL‐10* was performed to detect if compounds were acting as pro‐inflammatory (*IL‐8*) or antiinflammatory (*IL‐10*) agents. Primer sequences are presented in Table 2.

**Table 2:** Primers used in qRT‐PCR for quantification of reference and target genes in IPEC‐J2 cells

Gene	Accession no.	Sequence	Annealing T°
*CAT*	NM_214301	5′‐GTGCCAACGAAGATAATGTC 3′‐GACCCGCAATGTTCTCAC	63.0
*SDHA*	DQ845177	5′‐GCAGGCCAGGAGATAAAGTTC 3′‐GTTCCGTTCGCAAATCTCAG	63.0
*TBP*	DQ178129	5′‐GGTTTAGGTTGCAGCACCAG 3′‐CCAAATAGCAGCACAGTACGAG	56.0
*HSP70*	NM_001123127	5′‐CAGGTGCAAAGTACAAGACAAG 3′‐ATGGGACGACAAATCTGCT	63.0
*IKBA*	NM_001005150	5′‐CTGCACTTGGCCATCATC 3′‐GAGTCTGCTGCAGGTTGTTC	58.0
*IL‐8*	NM_213867.1	5′‐TCCTGCTTTCTGCAGCTCTC 3′‐GGGTGGAAAGGTGTGGAATG	62.0
*IL‐10*	NM_214041.1	5′‐ACCAGATGGGCGACTTGTTG 3′‐TCTCTGCCTTCGGCATTACG	63.0
*IL‐12*	NM_213993	5′‐GGCCTGCTTACCACTTGAAC 3′‐GCATTCATGGCCTGGAACTC	61.0

Total RNA was isolated according to the manufacturer's protocol (Promega, Madison, WI, USA) and quantified by spectrophotometer (Nanodrop ND‐100, Thermo scientific). Subsequently, 1 µg of extracted total RNA was reverse transcribed with the iScriptTM cDNA Synthesis kit (BIO‐RAD, Hercules, CA, USA). The obtained cDNA was diluted to a final concentration of 30 ng/μL. Primers were commercially produced (Eurogentec, The Netherlands) and were selected based on specificity and efficiency by qPCR analysis of a dilutions series of pooled cDNA at a temperature gradient (55 °C to 65 °C) for primer‐annealing and subsequent melting curve analysis. The reaction mixture for the qPCR containing 10 μL of the diluted cDNA was mixed with 15‐μL iQSYBR Green Supermix (Bio Rad Laboratories Inc., USA), forward and reverse primers (final concentration of 0.4 pmol/μL for each primer) and sterile water according to the manufacturer's instructions.

qRT‐PCR was performed using the MyIQ single‐colour real‐time PCR detection system (Bio‐rad, Hercules, CA) and MyiQ System Software Version 1.0.410 (Bio Rad Laboratories Inc., USA). The qRT‐PCR conditions were 1 min at 95 °C followed by 40 primer annealing cycles at the best melting curve temperature, and a final extension at 72 °C for 10 min. PCR products were bound using SYBR Green Supermix (Bio‐Rad), and the fluorescent signals were compared to arrive at the relative expression compared to housekeeping genes catalase (*CAT*), succinate dehydrogenase (*SDHA*) and tata‐binding protein (*TBP*). Amplification efficiency was determined per plate using linreg PCR (Ruijter *et al*., 2009). Data were analysed using the efficiency corrected Delta‐Delta‐Ct method (Pfaffl, 2001). The fold‐change values of the genes of interest were normalized using the geometric average of the fold‐change values of multiple housekeeping genes.

### Identification of secreted proteins

In order to assess the broader impact of the test compounds on ST, a proteomic analysis was carried out according to the methods described by Holland *et al*. (2010). After growth of bacteria in all test cultures to late‐exponential phase, bacterial cells were removed by centrifugation at 3500 *g* for 10 min at 4 °C. Supernatants were filtered through a 0.22‐µm pore‐size membrane filter to remove residual bacteria. Extracellular proteins were precipitated with trichloroacetic acid (TCA). The filtrate (100 mL) was mixed with 25% TCA to a final concentration of 6% and incubated overnight at −20 °C. The mixture was centrifuged for 30 min (6000 × g and 4 °C), and the resulting pellet was resuspended in 1 mL of acetone. The mixture was centrifuged for 15 min (20 000 × g and 4 °C), washed twice with acetone and the resulting pellet was air dried. The pellets were dissolved in PBS buffer and Laemmli sample before analysed by SDS PAGE.

The SDS‐PAGE separated sample was prepared for MS analysis by in‐gel digestion using trypsin. The tryptic peptides were micro‐purified using C18 stage tips according to the manufacturer's instructions (Proxeon, Thermo Scientific). NanoESI‐MS/MS analyses were performed on an EASY‐nLC II system (ThermoScientific) connected to a TripleTOF 5600+ mass spectrometer (AB Sciex) equipped with a NanoSpray III source (AB Sciex) operated under Analyst TF 1.5.1 control. The trypsin digested samples were suspended in 0.1% formic acid, injected, trapped and desalted on a trap column (Biosphere C18 column, 5 µm, 2 cm × 100 µm I.D; Nano Separations) after which the peptides were eluted onto and separated by an analytical column. Peptides were eluted using 250 μL/min and a 50 min gradient from 5% to 35% phase B (0.1% formic acid and 90% acetonitrile).

Proteins were identified using MASCOT (http://www.matrixscience.com) allowing a peptide mass tolerance of 100–300 ppm and ±0.3 Da for the fragment mass tolerance. Search parameters were: one missed trypsin cleavage site; propionamide as a fixed modification and methionine oxidation as variable modification. As search database, we used all proteins derived from *Salmonella* spp. that are stored in UniProt (http://www.uniprot.org/).

The experiments were not blinded or randomized except where stated.

### Statistical analysis

Data were compared by one‐way ANOVA followed by a post hoc test of least significant difference using SPSS (version 16.0) software. All probabilities are quoted as significant at the 5% level. For qRT‐PCR test, down‐ and up‐regulation were considered significant when the relative expression was decreased or increased >two‐fold.

## Results

### MBC, MIC, NIC and interactions between cinnamaldehyde, carvacrol and organic acids

The MBC, MIC and NIC were determined by incubation in an automated optical density meter and plating out on agar. The results of these assays are presented in Table 1. The MBCs for cinnamic acid and lactic acid were equal to the MIC. A checkerboard assay to screen for interactions between the compounds was also carried out; these results are presented in Table 3. No synergy or antagonism was found between cinnamaldehyde or carvacrol and the acids.

**Table 3:** No synergy or antagonism was detected between cinnamaldehyde or carvacrol with cinnamic, lactic or propionic acid

	Test compounds			
	Cinnamaldehyde		Carvacrol	
	FIC Index*	Interaction	FIC Index	Interaction
Cinnamic acid	0.7	Additive	1.3	Indifference
Lactic acid	1.0	Additive	1.1	Indifference
Propionic acid	1.0	Additive	1.2	Indifference

* FIC Index: Fractional Inhibitory Concentration Index *(0.5 – ±1.0 additive; >1 to <2 indifferent (*EUCAST, 2000 *))*

#### Effect of cinnamaldehyde, carvacrol and organic acids on motility of ST

**Figure 1:**
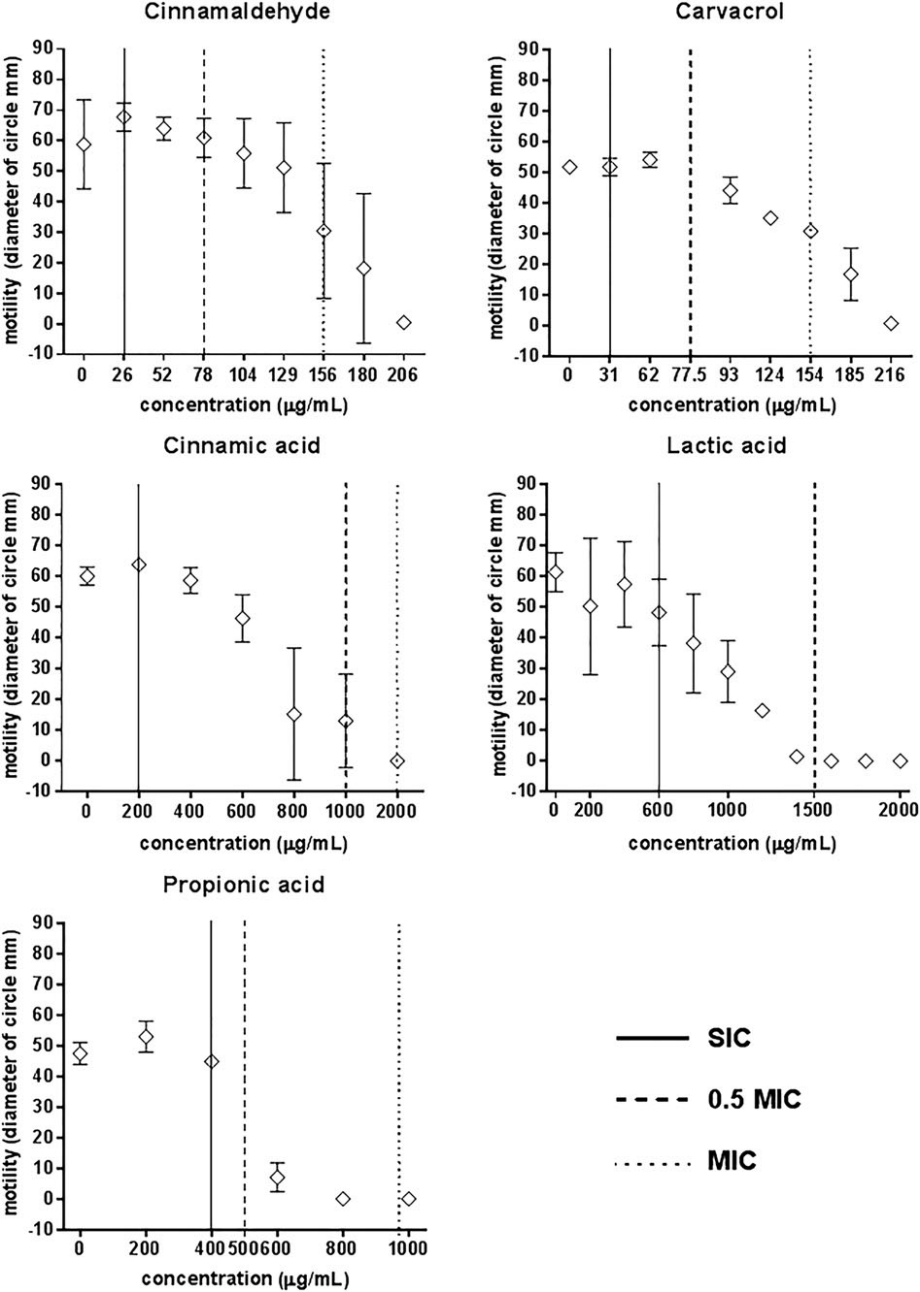
Cinnamaldehyde (a), carvacrol (b), cinnamic acid (c), lactic acid (d) and propionic acid (e) do not affect motility of *S. typhimurium* ATCC 14028 at NIC. Data points represent the mean of four independent experiments in which samples were carried out in duplicate.

To evaluate the effect of cinnamaldehyde, carvacrol and cinnamic, lactic, propionic acids on the motility of ST, 16‐h cultures were centrally inoculated onto soft agar plates containing a range of concentrations of each compound. The experiment was carried out three times on different days. The results of the motility measurements are presented in Fig. 1. At 0.5 × MIC neither cinnamaldehyde nor carvacrol reduced motility but cinnamic, lactic and propionic acids did. At the NIC, none of the compounds reduced motility.

### Viability of IPEC‐J2 cells is not affected by 0.5 × MIC of compounds

**Figure 2:**
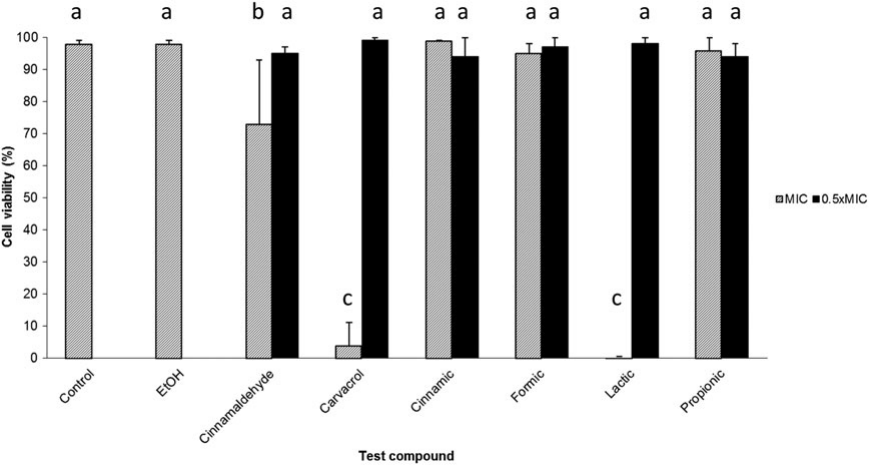
No compounds tested at 0.5 × MIC reduced viability of IPEC‐J2 cells after 2‐h contact. Ethanol as solvent control. Bars indicate standard deviation. Data points represent the mean of three independent experiments in which samples were carried out in duplicate.

The effect of the MIC and 0.5 × MIC of the compounds on the viability of IPEC‐J2 cells was determined by trypan blue exclusion assay, and the results are presented in Fig. 2. Cinnamaldehyde, carvacrol and lactic acid markedly reduced viability at the MIC; the other compounds did not. None of the compounds affected cell viability after exposure to 0.5 × MIC.

### Sub‐lethal concentrations of cinnamaldehyde, carvacrol and organic acids affect invasion of IPEC‐J2 cells by ST

**Figure 3:**
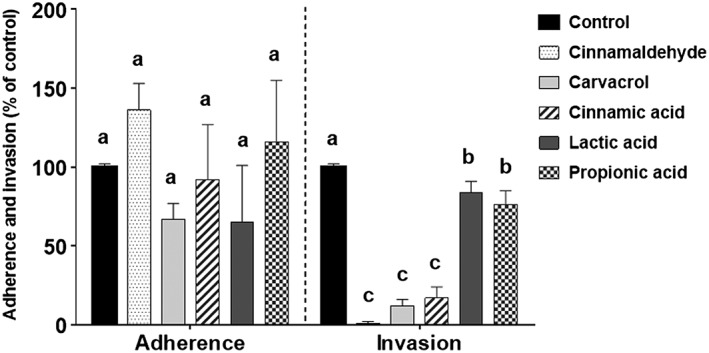
Invasion of IPEC‐J2 by Salmonella is reduced by sub‐lethal concentrations (0.5 × MIC) of cinnamaldehyde, carvacrol, cinnamic acid and propionic acids, although no compound affects bacterial adherence. Error bars indicate standard error. Data points with different letters are significantly different (*p* < 0.05). Data points represent the mean of three independent experiments in which samples were carried out in triplicate.

Assays were carried out using IPEC‐J2 cells as a model of the surface of the porcine intestine. The effect of 0.5 × MIC concentrations of cinnamaldehyde, carvacrol and cinnamic, lactic and propionic acids on the adherence to and invasion of IPEC‐J2 cells by *S. typhimurium* is presented in Fig. 3. No compound significantly influenced *S. typhimurium* adherence, but all compounds significantly reduced invasion compared to the control (*p* < 0.05). Cinnamaldehyde, carvacrol and cinnamic acid reduced invasion significantly more than lactic and propionic acids (*p* < 0.05).

**Figure 4:**
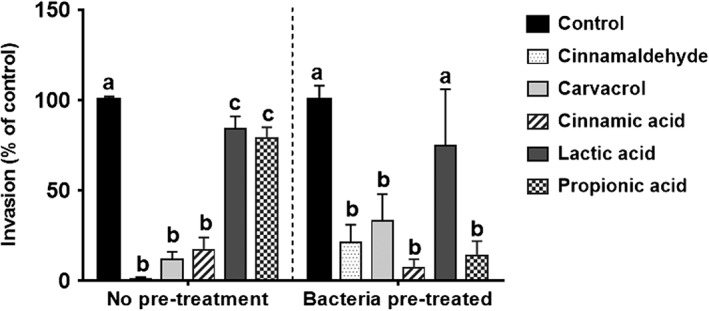
Pre‐treatment of *S. typhimurium* ATCC 14028 with NIC of compounds prior to the invasion assay reduced invasion of IPEC‐J2 cells further for propionic acid but not for the other compounds. Error bars indicate standard error. Data points with different letters are significantly different (*p* < 0.05). Data points represent the mean of three independent experiments in which samples were carried out in duplicate.

Although ST initially adheres to the cell membrane prior to invading the host cell, infection can only be achieved by invasion. In an experiment designed to more closely represent the situation in the porcine gut when additives are added to the feed, invasion assays were repeated using ST cultured in lower concentrations (i.e. NIC) of test compounds (see Table 1). This adjustment to the assay method also avoided any reduction of bacterial motility by the three acids, which inhibited motility at 0.5 × MIC (as indicated in Fig. 1). The results are presented in Fig. 4. Culture (pre‐treatment) of bacteria with cinnamaldehyde, carvacrol, cinnamic acid or lactic acid did not make a significant further difference to the inhibition of ST invasion of IPEC‐J2 cells achieved by the presence of the compounds in the cell culture medium. However, pre‐treatment with propionic acid caused a significant further reduction in ST invasion (*p* < 0.05).

**Figure 5:**
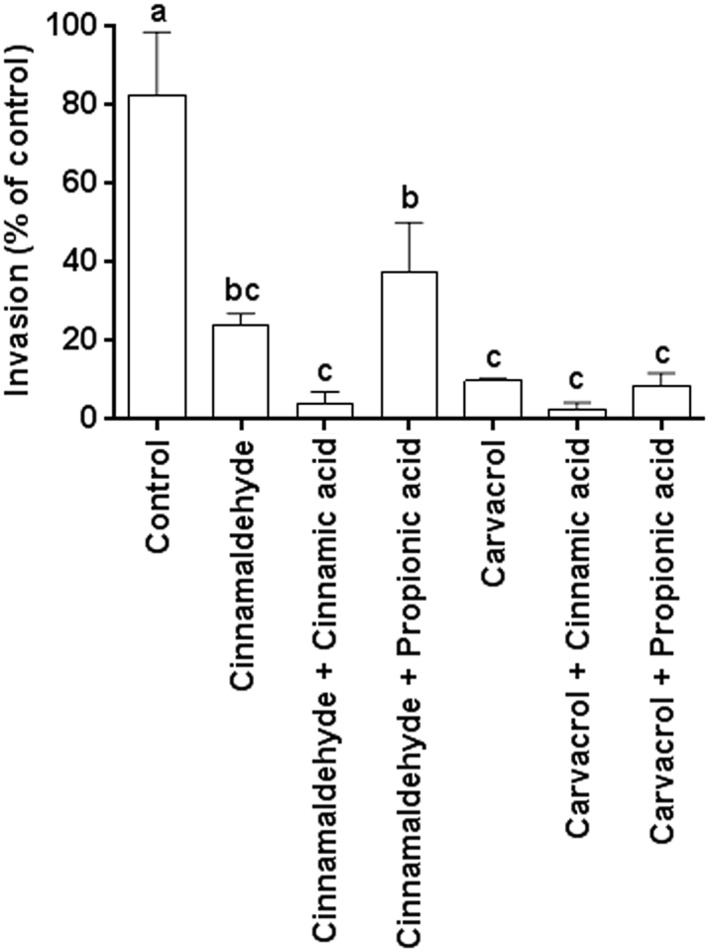
The presence of cinnamaldehyde, carvacrol, cinnamic and propionic acids at NIC during bacterial culture and invasion assays reduced invasion of *S. typhimurium* ATCC 14028 into IPEC‐J2 cells significantly. Combining cinnamaldehyde or carvacrol with the acids produced a further reduction of bacterial invasion in the case of carvacrol + propionic acid only. Bars indicate standard error. Data points with different letters are significantly different (*p* < 0.05). Data points represent the mean of three independent experiments in which samples were carried out in duplicate.

A further invasion assay was carried out using combinations of cinnamaldehyde or carvacrol with the organic acids, in which all compounds were applied at their NIC. Lactic acid was omitted because it had the weakest anti‐invasive properties in the earlier assays. The results are presented in Fig. 5. Combining cinnamaldehyde with cinnamic acid reduced *Salmonella* invasion significantly more than was achieved using cinnamaldehyde or cinnamic acid alone (*p* < 0.05). Adding propionic acid to cinnamaldehyde achieved no further reduction in invasion. For carvacrol, neither cinnamic nor propionic acids reduced invasion further (Fig. 5).

### Cinnamaldehyde, carvacrol and organic acids cause changes in fimbrial structure of ST

**Figure 6:**
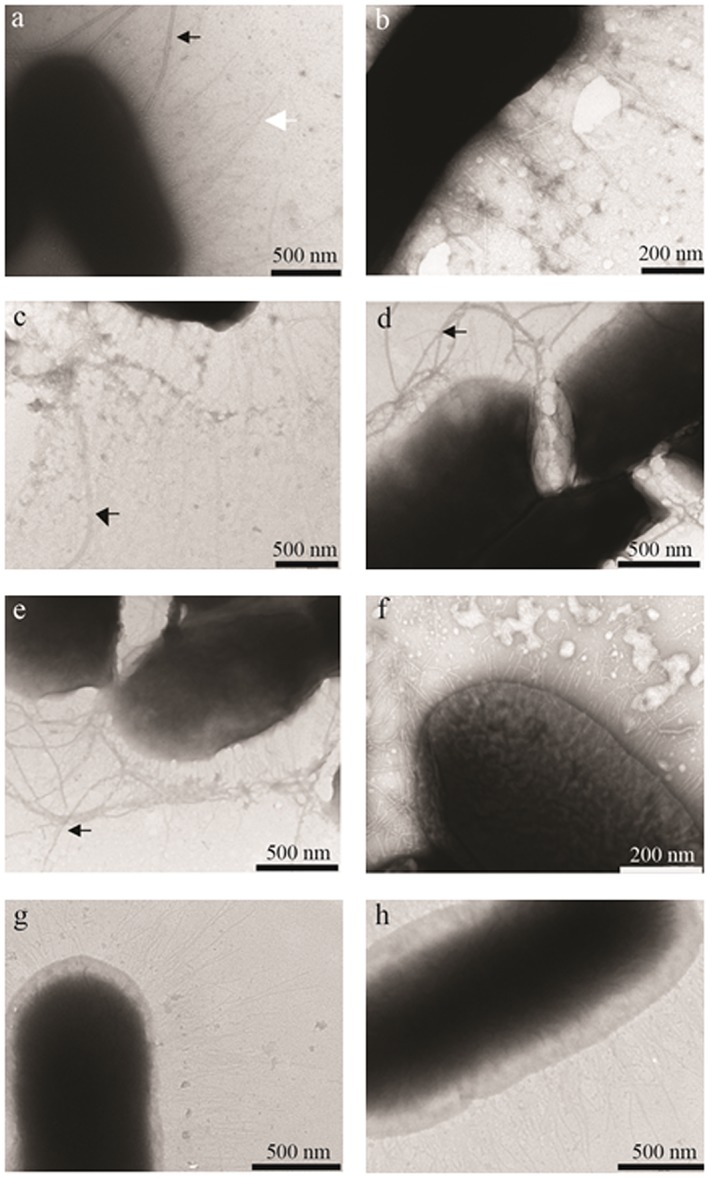
Electron micrographs of *S. typhimurium* ATCC 14028 cultured in LB with and without added test compounds at NIC. a: control (showing fimbriae, white arrow and flagella, black arrow), b: 26 µg/mL cinnamaldehyde, c: 31 µg/mL carvacrol, d: 200 µg/mL cinnamic acid, e: 600 µg/mL lactic acid, f: 400 µg/mL propionic acid, g: cinnamaldehyde + cinnamic acid and h: cinnamaldehyde + propionic acid. Flagella are indicated by a black arrow.

After growth in the presence of the NIC of the test compounds individually and in combination, bacteria were observed under an electron microscope, and the presence and structure of type 1 fimbriae were compared with untreated controls. Ethanol vehicle controls were indistinguishable from untreated controls. The images are presented in Fig. 6 (a – h).

ST cultured in control broth show bristle‐like structures typical for type 1 fimbriae (Fig. 6a). These structures are also visible on bacteria cultured in cinnamaldehyde, carvacrol, cinnamic acid and lactic acid (Figs. 6b–e). The fimbriae of bacteria grown in the presence of propionic acid appeared to be brittle and broken (Fig. 6f). Bacteria cultured in broth containing a combination of cinnamaldehyde with either cinnamic acid or propionic acid possessed fimbriae which were thinner than normal (Figs. 6g–h). Flagella were visible on all samples and are indicated on several images in Fig. 6.

### Cinnamaldehyde, carvacrol and organic acids affect mRNA relative expression of immune related genes in IPEC‐J2 cells

**Figure 7:**
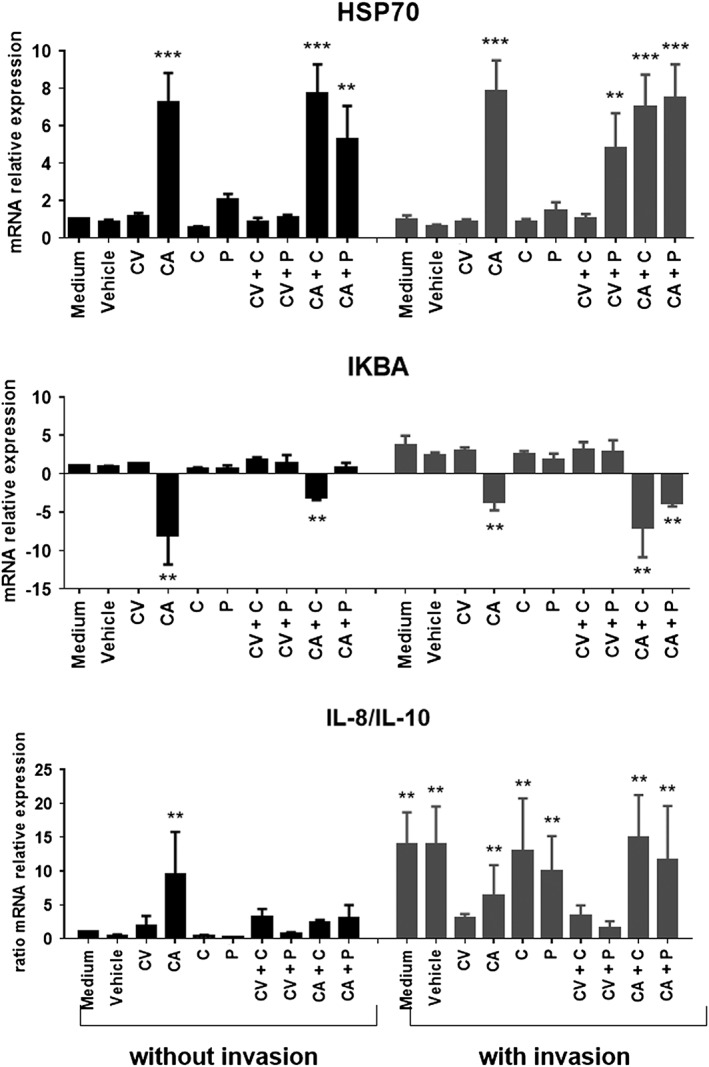
Cinnamaldehyde, carvacrol and organic acids affect mRNA relative expression of immune related genes in IPEC‐J2 cells. Incubation was performed with (grey bars) or without (black bars) bacterial exposure. Data points represent the mean of three independent experiments in which samples were carried out in duplicate. Legend: CV, carvacrol; CA, cinnamaldehyde; C, cinnamic acid; P, propionic acid.

The expression levels for selected genes associated with the oxidative stress and immune response in IPEC‐J2 cells were analysed by qRT‐PCR to investigate whether the presence of cinnamaldehyde, carvacrol, cinnamic or propionic acids affected the relative expression of these genes in the presence and absence of ST. The selected genes were *HSP70*, *IkBα*, *IL‐8*, *IL‐10* and *IL‐12*. The results of the qRT‐PCR analysis are presented in Fig. 7. The expression level of the genes in the presence of the ethanol control was the same as the untreated control in all cases, indicating that the solvent had no effect on the results.

HSPs are chaperones involved in the maintenance of protein structure in the cell under stress, and one of the most prominent marker for stress is the HSP70. Therefore, HSP can be a marker to evaluate the stress not only caused by bacterial invasion, but also caused by the test compound itself. *HSP70* was up‐regulated in IPEC‐J2 cells exposed to cinnamaldehyde regardless whether it was combination with cinnamic or propionic acid, and independent of bacterial invasion. *HSP70* mRNA expression was increased by bacterial invasion only when cells were exposed to the combination of carvacrol and propionic acid.

*IkBα* was down‐regulated in IPEC‐J2 cells exposed to cinnamaldehyde with or without cinnamic acid independent of bacterial invasion. *IkBα* was also down‐regulated in IPEC‐J2 cells exposed to cinnamaldehyde combined with propionic acid when cells were subjected to invasion. Although it was observed an up‐regulation (~two–four‐folds increase) in the mRNA expression of *IkBα* when IPEC‐J2 were exposed to *Salmonella* alone or in medium supplemented with carvacrol, cinnamic acid or propionic acid, either singly or combined, no significant differences were observed. It is known that in most cells NF‐kB can be found inactive in the cytoplasm when bound to IkB. Under pro‐inflammatory stimulation, activators such as interferon alpha and interleukins will cause a proteasomal degradation of IkBs, especially IkBa (Karin, 1999). It is expected that some compounds are able to inhibit this degradation, and the phosphorylated form of IkBa can accumulate to inactivate NFkB. However, in the present study, we have observed a tendency of IkBa up‐regulation, but it was not different from not treated cells.

IL‐12 mRNA expression was under the detection limit, thus not measured. IL‐8/IL‐10 ratio increased in IPEC‐J2 exposed to cinnamaldehyde even without bacterial invasion. Bacterial invasion of IPEC‐J2 cells led to an increased IL‐8/IL‐10 ratio, which was decreased only when cells were exposed to carvacrol alone or combined with propionic or cinnamic acid.

### Cinnamaldehyde, carvacrol and organic acids affect the secretome of ST

To assess the broader impact of the test compounds on the biological response of ST under all test and control conditions, an analysis of the extracellular proteome was performed. The results are presented in Table 4.

**Table 4:** Proteins identified in the extracellular fractions from control and treated *Salmonella*

Score*		Matches	% cov.	Accession	Annotation	Protein groups
Control						
479		19	30	A0A021WIC0	Flagellin	Flagellin
CA**						
514		12	25.3	A0A021WIC0	Flagellin	Flagellin
109		2	2.5	A0A021WVG0	Flagellar hook‐associated protein 1	Hook‐associated proteins
56		1	3.3	A0A021WPY5	Elongation factor Tu	Nucleotide binders
53		2	3.2	A0A021WSJ2	Flagellar hook‐associated protein 2	Hook‐associated proteins
52		2	2.6	A0A0F2YRW9	Uncharacterized protein	Uncharacterized
47		1	6.6	A0A021WUE6	Flagellar hook‐associated protein FlgL	Hook‐associated proteins
CV						
245		5	8.3	A0A021WIC0	Flagellin	Flagellin
46		1	78.3	Q9R4W8	Type 1 fimbrillin	Cell surfaces, metal and sugars binders***
36		1	3.2	A0A021WSJ2	Flagellar hook‐associated protein 2	Hook‐associated proteins
35		1	1.4	A0A0H3SK56	Tail sheath protein	Cell surfaces, metal and sugars binders***
34		1	2.7	A9MRT2	ATP‐dependent dethiobiotin synthetase BioD	Nucleotide binders
C ac						
	181	3	2.4	A0A021WIC0	Flagellin	Flagellin
L ac						
154		3	2.4	A0A021WIC0	Flagellin	Flagellin
	31	2	3.4	V1GXD3	IbrA protein	Catalytic proteins
P ac						
165		3	2.4	A0A021WIC0	Flagellin	Flagellin
	58	1	14.8	G5N7D6	Ferredoxin‐like protein FixX	Cell surfaces, metal and sugars binders***
CA + C ac						
161		5	7.7	A0A021WIC0	Flagellin	Flagellin
	60	1	3.2	A0A021WSJ2	Flagellar hook‐associated protein 2	Hook‐associated proteins
CA + P ac						
153		4	5.3	A0A021WIC0	Flagellin	Flagellin
	67	1	3.2	A0A021WSJ2	Flagellar hook‐associated protein 2	Hook‐associated proteins
CV + C ac						
135		3	2.4	A0A021WIC0	Flagellin	Flagellin
	51	1	10.8	A0A021WR18	Type‐1 fimbrial protein subunit A	Cell surfaces, metal and sugars binders***
CV + P ac						
367		15	30	E7DXY1	Flagellin	Flagellin
167		5	18.4	A0A021WR18	Type‐1 fimbrial protein subunit A	Cell surfaces, metal and sugars binders***
159		7	8.6	A0A021WPY5	Elongation factor Tu	Nucleotide binders
125		5	6.6	A0A021WSJ2	Flagellar hook‐associated protein 2	Hook‐associated proteins
114		2	78.3	Q9R4W8	Type 1 fimbrillin	Cell surfaces, metal and sugars binders***
58		3	4.5	A0A0C8R7N1	Elongation factor G	Nucleotide binders
53		1	2.5	A0A021WVG0	Flagellar hook‐associated protein 1	Hook‐associated proteins
46		1	3.9	A0A0J6DBW9	D‐tagatose‐1,6‐bisphosphate aldolase subunit KbaY	Catalytic proteins
44		3	6.6	P16326	Flagellar hook‐associated protein 3	Hook‐associated proteins
35		1	2.4	A0A0G3B0P1	TnpA	Cell surfaces, metal and sugars binders***
34		1	13.7	G4BXA1	Uncharacterized protein	Uncharacterized

* The listed proteins are sorted according to the Mascot score.

** CA = cinnamaldehyde, CV = carvacrol, C ac = cinnamic acid, L ac = lactic acid, P ac = propionic acid.

*** Pathogenicity associated.

Flagellin was detected in the secretome of all samples independent of the treatment. However, this protein was detected in the control group with relatively high coverage, whereas in most treated samples, the flagellin coverage was low. Besides flagellin and other flagellum proteins, only a few other proteins were detected in the supernatant fraction such as fimbrial proteins. Importantly, differences in flagellum and fimbriae proteins were detected in most treated bacteria compared to the control. It was observed that the most efficient treatments, i.e. cinnamaldehyde + cinnamic acid, cinnamaldehyde + propionic acid, carvacrol, carvacrol + cinnamic acid and carvacrol + propionic acid, resulted in the detection of flagellar hook and fimbrial proteins in the supernatant. This may indicate that the flagellar apparatus of *Salmonella* is affected and that the adhesion properties mediated by fimbriae are modified by these treatments. The increase in the secretion of fimbrial proteins correlates with increased inflammation of the affected cells.

## Discussion

The aim of this study was to assess the effect of selected phytochemicals and organic acids, alone and in combination, on the invasion of ST into porcine gut cells. Our findings show that carvacrol and cinnamaldehyde inhibit invasion of ST into gut epithelial cells by means other than toxicity to bacteria or cells, and that this effect is increased in the presence of certain organic acids. The findings also show a potential basis for this activity in the expression levels of immune‐related genes by the cells.

There are some limitations when using the monoculture IPEC system when compared with the complexity of the gut. Importantly, the natural microflora of the gut is absent, as is the link to a systemic immune reaction. There is no interaction with the test compounds by gut flora or digesta and other factors found only in vivo are also absent. However, the non‐transformed continuous IPEC‐J2 cell line has been widely used as a model for the porcine and human gut over more than a decade (Zakrzewski *et al*., 2013) and, in view of the 3R approach, the IPEC‐J2 cell line is a useful first step in screening diet additives before progressing to animal feeding trials.

The MIC we determined for cinnamaldehyde (156 µg/mL) is lower than that reported for *S. typhimurium* SG1 (2.5 mM or 325 µg/mL) (Palaniappan and Holley, 2010). The MIC for carvacrol determined in this study (154 µg/mL) is slightly lower than that determined by others for the same bacterial strain (0.025% or 250 µg/mL) (Soni *et al*., 2013), where the use of a richer growth medium (tryptic soy broth) may have contributed to this difference. Other strains of ST have identical MICs for carvacrol (Nazer *et al*., 2005) or appear less sensitive to the natural antimicrobials used here; the MIC for carvacrol was 2 mM (308 µg/mL) for *S. typhimurium* DT104 and 2.5 mM (385 µg/mL) for *S. typhimurium* SG1 (Inamuco *et al*., 2012; Palaniappan and Holley, 2010). The MBCs of cinnamaldehyde and carvacrol in our hands confirm earlier reports for the same bacterial strain (Mith *et al*., 2014). There is little data reported for interactions between cinnamaldehyde and carvacrol and these acids. A very weak indication for synergy was found between carvacrol and lactic acid for ST ATCC 13311 (Kobilinsky *et al*., 2007).

At the concentrations used in the adherence and invasion assays, 0.5 × MIC and the NIC, no effect on bacterial motility was observed for cinnamaldehyde or carvacrol (Table 1 and Fig. 1). Therefore, any influence on the invasive capacity of ST by these compounds (Figs. 3, 4, 5) cannot stem from inhibition of bacterial motility. For carvacrol, these results are in accordance with a previous study using *S. typhimurium* DT104 (Inamuco *et al*., 2012) and may be associated with disruption of bacterial quorum sensing (Burt *et al*., 2014).

A report for *Salmonella enteritidis* found that the NIC of cinnamaldehyde led to lower expression of *motA* and therefore reduced motility (Kollanoor‐Johny *et al*., 2012). However, the NIC of cinnamaldehyde (0.01%, approx. 100 µg/mL) was four‐fold that used in the present study so it may be more likely that expression of *motA* is influenced.

The three acids reduced motility at 0.5 × MIC, which may explain the slight reduction in invasion at these concentration as shown in Fig. 3. It was to avoid this effect that we chose to repeat the invasion assay at lower concentrations (SIC) and these results are presented in Fig. 5.

IPEC‐J2 cell viability was not adversely affected by exposure to 0.5 × MIC of the test compounds (Fig. 2). Therefore, reduced cell viability cannot have contributed to the observed reductions in adherence and invasion by ST (Figs. 3, 4, 5).

Our results for carvacrol confirm other studies that show that sub‐lethal concentrations can reduce invasion of *S. typhimurium* DT104 into IPEC‐J2 cells (Inamuco *et al*., 2012), *S. enteritidis* into chicken oviduct epithelial cells (Upadhyaya *et al*., 2013) and reduce invasion of *C. jejuni* into INT407 cells (Van Alphen *et al*., 2012). The inhibition of bacterial attachment and biofilm formation by carvacrol at concentrations around 0.2 mM (31 µg/mL) has recently been associated with disruption of bacterial quorum sensing (QS) (Burt *et al*., 2014). However, in the present study, although carvacrol inhibited *Salmonella* invasion significantly, the NIC of carvacrol (31 µg/mL) did not adversely affect bacterial motility (Fig. 1) or flagella formation (Fig. 6c), both of which are associated with QS (Merighi *et al*., 2009). Sajed *et al* (2013) reviewed the immuno‐stimulatory effects of essential oil containing carvacrol.

This appears to be the first report to show that cinnamaldehyde reduces ST invasion of epithelial cells. The NIC of cinnamaldehyde for *S. enteritidis* (0.01% *v/v*, approx. 100 µg/mL), although much higher than the NIC for ST in our hands (26 µg/mL, Table 1), was also found to reduce invasion of epithelial cells by approximately 60–80% in four strains of *S. enteritidis*, associated with reduced expression of invasion genes *hilA*, *hilD* and *invF* (Kollanoor‐Johny *et al*., 2012).

Inhibition of ST invasion into porcine intestinal (IPI‐2I) epithelial cells by propionic acid was also reported by Boyen *et al*. (2008) although the concentration used (10 mM) was approximately twice that used in the present study. Expression of the promoter region that regulates invasion genes, *hilA*, was down‐regulated by culture with 10 mM propionic acid (Boyen *et al*., 2008).

The presence of flagella appeared unaffected by culture in the presence of the NICs of the test compounds (Fig. 6). These observations are in accordance with earlier work on *S. typhimurium* ST104 (Inamuco *et al*., 2012). However, the physical structure of type 1 fimbriae with which salmonellae attach to epithelial cells prior to invasion was changed by culture in propionic acid and by cinnamaldehyde combined with cinnamic acid (Figs. 6f–h). It is proposed that the disrupted structure of the fimbriae contributed to the observed reduction of invasion capacity. Cinnamaldehyde, carvacrol and cinnamic acid caused no noticeable changes in the fimbrial structure (Figs. 6b–d). Because these compounds significantly reduced invasion of epithelial cells (Figs. 3, 4, 5), virulence factors other than fimbriae must have been affected by these compounds. A recent study examining fatty acid changes in *S. typhimurium* ATCC 14028 membranes found that 0.6 μL/mL (62 µg/mL) carvacrol in meat broth caused a shift towards synthesis of unsaturated fatty acids and *cis–trans* isomerization and sub‐lethal damage (Luz *et al*., 2014). However, the concentration used is approximately 10‐fold higher than used in the present study, possibly because of the protection of bacteria by proteins in the meat broth (Luz *et al*., 2014).

It is well known that HSPs are induced in response to stress conditions. We have observed that mRNA expression of *HSP70* was increased when cells were exposed to cinnamaldehyde alone or in combination with cinnamic or propionic acid. Also, such up‐regulation was not dependent on exposure to *Salmonella*. *HSP70* up‐regulation in cells exposed to *Salmonella* was observed only when carvacrol was used in combination with propionic acid. These findings may be an indication that cinnamaldehyde is a stressor for the cells at the used concentration and, probably, carvacrol with propionic acid activated *HSP70* to counteract bacterial invasion. Corroborating with our findings, Wieten *et al*. (2010) have shown that carvacrol is a potent *HSP70* up‐regulator, but not a stress inducer, and acts as an antiinflammatory agent. In the present study, bacterial invasion appeared to be not sufficient to cause oxidative stress, as no *HSP70* up‐regulation was observed in this treatment group. Therefore, we suggest that carvacrol was a *HSP70* co‐inducer when cells were stressed because of bacterial invasion in combination with the presence of the stressor propionic acid.

IkBα is a NF‐kB inhibitor, which needs to be degraded to activate NF‐kB. Therefore, *IkBα* down‐regulation is a signal of NF‐kB activation involved in cell response to stress or inflammation. It has been claimed that cinnamaldehyde has antiinflammatory properties by inhibiting pro‐inflammatory cytokines (Youn *et al*., 2008). However, in the present study, we have observed the contrary. The fact that *IkBα* was down‐regulated in IPEC‐J2 cells exposed to cinnamaldehyde with or without cinnamic acid corroborates with the up‐regulation of *HSP70* by those compounds. The inflammatory role of cinnamaldehyde was further confirmed by the increased ratio IL‐8/IL‐10. IkBα down‐regulation has also been reported in ulcerative colitis (Feng *et al*., 2012).

The antiinflammatory role of carvacrol has been linked to the induction of *IL‐10* (Lima *et al*., 2013), and this can be confirmed by the decreased *IL‐8/IL‐10* ratio when cells were infected and exposed to this compound in the present study. *Salmonella* elicits a strong inflammatory response 8 h after infection (Hapfelmeier *et al*., 2004). However, we exposed IPEC‐J2 cells to ST for 2 h. Also, *Salmonella* delivers effectors able to modulate host immune defence by interfering with NF‐kB activation (Jones *et al*., 2008; Valdez *et al*., 2009). It is possible that this explains why *IkBα* was not down‐regulated but tended to be up‐regulated, although the *IL‐8/IL‐10* ratio was already indicating cellular response to inflammation.

The analysis of the secretome revealed changes in the flagellar apparatus and irregularities in fimbrial proteins (Table 4) that correlated with the visual images of the treated bacteria (Fig. 2) and the reduced invasion of epithelial cells by ST (Figs. 4 and 5). This applies in particular to combinations of the phytocompounds with certain acids, particularly cinnamaldehyde with cinnamic acid and carvacrol with either cinnamic or propionic acid. The increase in the secretion of fimbrial proteins is correlated with increased inflammation of the affected cells (Kuźmińska‐Bajor *et al*., 2015). Ibarra *et al*. (2010) also showed that type 1 fimbriae production is increased as a bacterial stress response, and this production does not result in increased invasion, but it is a result of impaired ability to invade host‐cells.

### Clinical relevance of the results and further research

These findings reveal information about the mechanism behind the inhibitive effect of phytochemicals on ST invasion capacity. From the results of this study, the combination of carvacrol with propionic or cinnamic acid appears to inhibit ST invasion the most whilst causing inflammatory reaction in the host cells to a lesser degree than cinnamaldehyde. The physiological relevance of the results of these experiments has yet to be demonstrated in animal feeding trials.

## Conclusion

This study shows that cinnamaldehyde, carvacrol, cinnamic and propionic acids inhibit the ability of ST to invade IPEC‐J2 cells when present at concentrations too low to affect cell viability, bacterial growth or viability, bacterial motility or the development of flagella. In some cases, these low concentrations cause malformations of type 1 fimbriae and change the expression levels of some immune‐related genes in IPEC‐J2 cells. Use of essential oils and organic acids as novel antimicrobial agents has gained interest, and many compounds have presented antimicrobial activity. However, it is important to determine if these compounds have pro‐inflammatory properties, as well as if they can act as stressors also to the host of the bacteria. For instance, cinnamaldehyde induces inflammation and stress in the IPEC‐J2 cells. Carvacrol did not induce inflammation in the cells unless combined with propionic acid and on exposure to ST.

## Conflict of Interest

No ethics clearance was required.
